# Impact of Concomitant Aberrant CD200 and BCL2 Overexpression on Outcome of Acute Myeloid Leukemia: A Cohort Study from a Single Center

**DOI:** 10.4274/tjh.galenos.2021.2020.0728

**Published:** 2021-06-01

**Authors:** Mario Tiribelli, Angela Michelutti, Margherita Cavallin, Sara Di Giusto, Renato Fanin, Daniela Damiani

**Affiliations:** 1 University of Udine, Department of Medical Area, Division of Hematology and Stem Cell Transplantation, Udine, Italy

**Keywords:** CD200, BCL2, Acute myeloid leukemia, Prognosis, Survival

## Abstract

**Objective:**

CD200 and BCL2 overexpression is independently associated with inferior survival in acute myeloid leukemia (AML), and these two factors are frequently co-expressed; however, no data are available on the role of concomitant aberrant CD200 and BCL2 expression on outcome of AML patients. We aimed to elucidate the prognostic role of CD200/BCL2 co-expression and its association with specific leukemia subsets.

**Materials and Methods:**

We analyzed 242 adult AML patients uniformly treated with intensive chemotherapy, evaluating the impact of CD200 and BCL2 expression on complete remission (CR), disease-free survival, and overall survival (OS).

**Results:**

CD200 and BCL2 were expressed in 139 (57.4%) and 137 (56.6%) cases, respectively, with 92 patients (38%) displaying double positivity (DP), 58 (24%) displaying double negativity (DN), and 92 patients expressing only either CD200 (n=47) or BCL2 (n=45). CR was achieved in 71% of cases, being less frequent in DP patients (60%) compared to other groups (76%-81%, p<0.001). In the whole population 3-year OS was 44%, being lower in DP patients (28%) than in patients with single CD200 or BCL2 expression (47%) or DN cases (60%; p=0.004). Other factors associated with worse OS were advanced age, CD34 positivity, secondary AML, and high white blood cell count at diagnosis; combining these 4 factors with CD200/BCL2 DP, we identified 6 groups with significantly different rates of survival (3-year OS ranging from 90% to 0%).

**Conclusion:**

Our data support a synergistic effect of CD200 and BCL2 in AML cells, conferring an enhanced survival capacity in a permissive microenvironment and resulting in worse prognosis.

## Abstract (tr)

**Amaç:**

CD200 ve BCL2 aşırı ekspresyonu, bağımsız olarak, akut myeloid lösemide (AML) düşük hayatta kalma ile ilişkilidir ve bu 2 faktör sıklıkla birlikte ifade edilir; bununla birlikte, AML hastalarının akıbeti üzerindeki eşzamanlı anormal CD200 ve BCL2 ekspresyonunun rolü hakkında hiçbir veri mevcut değildir. CD200/BCL2 birlikte ifadesinin prognostik rolünü ve bunun spesifik lösemi alt kümeleri ile ilişkisini aydınlatmayı amaçladık.

**Gereç ve Yöntemler:**

CD200 ve BCL2 ekspresyonunun tam remisyon (CR), hastalıksız sağkalım (DFS) ve genel sağkalım (OS) üzerindeki rolünü değerlendirerek, yoğun kemoterapi ile eşit şekilde tedavi edilen 242 yetişkin AML hastasını analiz ettik.

**Bulgular:**

CD200 ve BCL2 sırasıyla 139 (%57,4) ve 137 (%56,6) olguda ifade edildi; 92 hastada (%38) çift pozitiflik (DP), 58 (%24) hastada çift negatiflik (DN) ve 92 hastada sadece CD200 (47) veya sadece BCL2 (45) ifadesi vardı . Hastaların %71’inde CR elde edildi, CR, DP hastalarında (%60) diğer gruplara göre (%76-81, p<0,001) daha düşüktü. Tüm popülasyonda 3 yıllık OS %44 olup, DP hastalarında (%28), tek CD200 veya BCL2 ekspresyonu olan hastalara (%47) göre ve DN olgularına göre (%60; p=0,004) daha düşüktür. Daha kötü OS ile ilişkili diğer faktörler ileri yaş, CD34 pozitifliği, ikincil AML ve tanıda yüksek WBC idi; Bu 4 faktörü CD200/BCL2 DP ile birleştirerek, önemli ölçüde farklı sağkalıma sahip 6 grup belirledik (3 yıllık OS %90 ila 0 arasında değişir).

**Sonuç:**

Verilerimiz, CD200 ve BCL2’nin AML hücrelerinde sinerjistik etkisini ve kısıtlanmamış bir mikro ortamda gelişmiş bir hayatta kalma kapasitesi sağlayarak daha kötü bir prognoza neden oluşu desteklemektedir.

## Introduction

The increased knowledge acquired in the last years on the genetic basis of acute myeloid leukemia (AML) has not yet translated into a significant increase in the curability of this tragic disease. Resistance to chemotherapy remains a major challenge and accounts for the high rate of disease recurrence experienced by most patients and, consequently, the disappointing long-term outcome of AML. It is well known that many factors are involved in poor response to treatment. Intrinsic disease characteristics confer an advantage to leukemic cells in terms of proliferation, expansion, and survival, in part due to reduced sensitivity to drugs or multidrug resistance [1,2,3,4] and in part due to the induction of a permissive microenvironment favoring the evasion of neoplastic clones from anti-tumor immune control [5,6,7,8,9]. The interest in the immunogenicity of leukemic cells has increased in parallel with the development of drugs able to inhibit or modulate the crosstalk between tumor cells and components of the innate or adoptive immune system, such as checkpoint inhibitors [10,11]. Moreover, in the past years, the role of the inhibition of apoptotic pathways, well known in lymphoproliferative disorders, has also emerged in acute leukemia [12,13,14,15,16,17]. Since we have observed a frequent association of BCL2 positivity and CD200 overexpression in AML patients [18], we aimed to elucidate the prognostic role of CD200/BCL2 co-expression and its association with specific leukemia subsets in our cohort of AML patients treated with intensive chemotherapy, with the purpose of identifying patients suitable for new targeted treatments.

## Materials and Methods

A total of 242 patients with non-promyelocytic AML, admitted between January 2009 and June 2018 to the Division of Hematology of the University of Udine, were included in the study. Cytological diagnosis was performed based on bone marrow and peripheral blood.

Multiparametric flow cytometry (FACSDiva II, BD) was employed to evaluate leukemia-associated antigens. CD200 was expressed as the percentage of positive cells (with 20% as a cut-off value) and as the mean fluorescence intensity (MFI) obtained by the ratio of fluorescence intensity of the test and its isotypic control. Cases with MFI =1 were considered negative, MFI <11 as low expression, and MFI ≥11 as high expression. BCL2 was considered overexpressed for MFI ≥17 (i.e., above the median value of the population). Cytogenetic risk was classified according to Medical Research Council criteria [19]. FLT3 and NPM1 mutations were evaluated as previously described [20].

All patients received intensive induction chemotherapy based on fludarabine, cytarabine, and idarubicin, followed by at least one consolidation course of high-dose cytarabine. In high-risk cases (defined by at least one of the following: secondary AML, poor response to induction chemotherapy, unfavorable cytogenetic or combined genetic risk, early relapse), allogeneic stem cell transplantation from a related or unrelated donor was pursued as post-induction therapy.

### Statistical Analysis

Complete remission (CR) was defined as complete peripheral hematological recovery and the absence of bone marrow disease upon morphological, immunophenotypic, or molecular evaluation. Overall survival (OS) was calculated from diagnosis to death (irrespective of its cause). Disease-free survival (DFS) was defined as the time from the date of CR to the date of relapse of AML. Patients lost to follow-up were censored at the time last seen alive.

Categorical variables were compared with the Fisher exact test or Yates corrected chi-square test as required. Factors affecting CR were assessed by univariate and multivariate logistic regression and expressed as odds ratios (ORs) with 95% confidence intervals (CIs). Survival curves were constructed by the Kaplan-Meier method and differences among groups were calculated by log-rank test. The Cox proportional hazard regression model was used to examine the potential prognostic factors for survival; all variables with values of p≤0.10 in univariate analysis were included in the multivariate model and a forward procedure was applied to identify significant factors.

Statistical analysis was performed with NCSS 11 Statistical Software (NCSS Statistical Software, Kaysville, UT, USA). All p-values are 2-sided at a significance level of 0.05.

## Results

The median age of the whole population was 58 (range: 18-81) years, and 143/242 (59%) patients were older than 55 years. One hundred ten patients (45.5%) were female and 132 (55.5%) were male. Seventy-one patients (29%) had secondary AML, developed from an antecedent myeloproliferative disease or after chemotherapy for a solid or hematologic neoplasm. Eighty-nine patients (37%) had high white blood cell (WBC) counts at diagnosis, defined as ≥30x10^9^/L, while the karyotype was favorable in 14 (6%), intermediate in 154 (64%), unfavorable in 61 (25%), and not evaluable in 13 (5%) cases, respectively.

CD200 was expressed in 139/242 cases (57.4%) and BCL2 was overexpressed in 137/242 (56.6%) cases. CD200 positivity and concomitant BCL2 overexpression (double positivity, DP) was found in 92/242 (38%) patients, while 58 (24%) revealed double negativity (DN) and 92 expressed only CD200 (47, 19.4%) or BCL2 (45, 18.6%).

Clinical and biological characteristics of patients according to CD200 and BCL2 expression are summarized in Table 1. CD200/BCL2 DP cases were more frequent among patients with WBC counts of <30x10^9^/L (64/92, 69.5% vs. 79/150, 56.5%; p=0.04), in CD34+ AML (57/92, 62% vs. 64/150, 42.7% in CD34-negative patients; p=0.004), and in cases of NPM1-wt (71/88, 80.7% vs. 73/130, 35.9% in NPM1 mutated cases; p<0.001).

### Response to Therapy

All patients received intensive induction chemotherapy and were evaluable for response. CR was obtained in 171/242 cases (70.7%).

In Table 2 factors affecting CR probability in univariate and multivariate analysis are listed. The CR rate was significantly lower in patients with CD200 expression (88/139, 63.3%) compared to CD200-negative cases (83/103, 80.6%; p=0.006), while BCL2 positivity was associated only with inferior CR rate (90/137, 65.7% vs. 81/105, 77.1%; p=0.07). According to CD200 and BCL2 expression, CR was reached by 55/92 (59.8%) DP patients, by 70/92 (76.1%) patients with either CD200 or BCL2 expression, and by 54/58 (81.0%) DN patients, resulting in a significantly lower CR rate among DP patients compared to all other groups (p<0.001). CR probability was also negatively affected by age of ≥55 years (89/143, 62.2% vs. 82/99, 82.8%; p=0.0005), secondary AML (33/71, 46.5% vs. 138/171, 80.7%; p<0.0001), unfavorable cytogenetics (33/61, 54.1% vs. 129/168, 76.8%; p=0.0008), CD34 positivity (69/121, 57% vs. 102/121, 84.3%; p<0.0001), and NPM1-wt (101/159, 63.5% vs. 64/74, 86.5%; p=0.0003).

In multivariate analysis statistical significance was maintained by advanced age (OR 2.1, 95% CI 1.07-4.21), secondary AML (OR 3.6, 95% CI 1.89-7.0), CD34 positivity (OR 3.1, 95% CI 1.62-6.0), and CD200/BCL2 DP (OR 1.9, 95% CI 1.03-3.7).

### Disease-Free Survival

At the time of analysis 61/171 patients had relapsed and 110/171 remained in CR, with a 3-year DFS of 60% (95% CI 10-68). Neither CD200 nor BCL2, alone or in association, influenced DFS. In univariate analysis an adverse effect on DFS was found only for unfavorable cytogenetics (3-year DFS 34%, 95% CI 17-52 vs. 65%, 95% CI 55-74 in other cytogenetics groups; p=0.001) and CD34 positivity (3-year DFS 46%, 95% CI 32-60 vs. 68%, 95% CI 58-78 in CD34-negative patients; p=0.01). Multivariate analysis confirmed their negative role for DFS, with a relapse risk of 1.4 (95% CI 1.04-2.94) for unfavorable cytogenetics and 2.6 (95% CI 1.17-3.5) for CD34 positivity.

### Overall Survival

**Figure 1:**
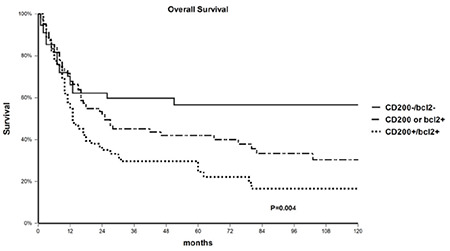
Overall survival of the entire population according to CD200 and BCL2 expression.

At the time of analysis, of 242 patients included in the study, 139 (57%) had died, with a 3-year OS in the whole population of 44% (95% CI 38-51). As shown in Table 3, in univariate analysis OS was negatively affected by age of ≥55 (3-year OS 34%, 95% CI 26-43 vs. 58%, 95% CI 48-68 in patients aged less than 55 years; p=0.0001), secondary AML (3-year OS 29%, 95% CI 16-41 vs. 50%, 95% CI 42-58 in de novo patients; p=0.0005), high WBC count at diagnosis (3-year OS 34%, 95% CI 24-45 vs. 51%, 95% CI 42-59 in patients with lower WBC counts; p=0.03), unfavorable cytogenetics (3-year OS 29%, 95% CI 17-41 vs. 49%, 95% CI 41-57 for favorable or intermediate karyotypes; p=0.003), NPM1-wt (3-year OS 39%, 95% CI 30-47 vs. 56%, 95% CI 44-68 in NPM1-mutated cases; p=0.005), and CD34 positivity (3-year OS 29%, 95% CI 20-38 vs. 59%, 95% CI 50-69 in CD34-negative cases; p<0.0001). Considering CD200 and BCL2, DP patients had a 3-year OS of 28% (95% CI 18-39) compared to 47% (95% CI 35-60) among patients with isolated CD200 or BCL2 overexpression and 60% (95% CI 46-73) among DN patients (p=0.004; Figure 1). In multivariate analysis, statistical significance was retained by age of ≥55 (OR 2.0, 95% CI 1.4-2.9), secondary AML (OR 1.58, 95% CI 1.07-2.32), high WBC count (OR 2.0, 95% CI 1.36-2.9), CD34 positivity (OR 2.2, 95% CI 1.5-3.2), and CD200/BCL2 DP (OR 1.5, 95% CI 1.05-2.1).

**Figure 2:**
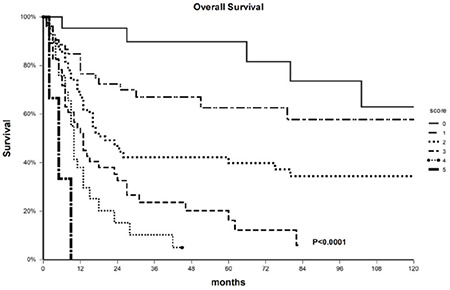
Overall survival by number of prognostic factors.

Finally, the five variables found significant by multivariate analysis were combined in a score predicting very different OS probabilities: 3-year OS was 90% (95% CI 76-100) for patients without risk factors, 67% (95% CI 53-80) for those with 1 risk factor, 42% (95% CI 31-54) for those with 2 risk factors, 25% (95% CI 11-38) for those with 3 risk factors, 10% (95% CI 0-23) for those with 4 risk factors, and 0% if all 5 risk factors were present (p<0.0001; Figure 2).

## Discussion

The dysregulation of several pathways compromising the differentiation ability or promoting proliferation and survival has been proposed for the development and clinical characterization of AML [21]. Overexpression of anti-apoptotic BCL2 family proteins resulting from chromosomal translocation, gene amplification, increased gene transcription, or alteration of post-transcriptional processing has been found for many solid and hematologic neoplasms [22,23]. In AML, high BCL2 expression has been associated with poor prognosis. Campos et al. [24] observed heterogeneous flow cytometric expression of BCL2 among 82 patients with de novo AML, but patients with >20% BCL2-positive leukemic cells had significantly lower CR rates and shorter survival. Karakas et al. [25] analyzed the BCL2 transcript in 152 patients with newly diagnosed AML, confirming a negative impact on CR, DFS, and OS. Del Poeta et al. described a negative impact on outcome by evaluating the ratio between pro-apoptotic BAX and anti-apoptotic BCL2 protein [17]. Mehta et al. [15] reported a significant reduction of DFS in patients with BCL2 overexpression and FLT3 internal tandem duplication. We previously reported a negative correlation between high BCL2 expression and OS and, for the first time, we observed a frequent association between BCL2 overexpression and aberrant expression of CD200 [18]. CD200 is a member of the immunoglobulin family expressed on the membrane of many cell types, such as thymocytes, activated T-cells, B-cells, dendritic cells, vascular endothelial cells, and central nervous cells. In humans CD200 exclusively binds to its inhibitory receptor, CD200R, physiologically acting as a regulator of the antimicrobial immune response controlling the return to homeostasis [26,27]. Moreover, there is a body of evidence suggesting that the CD200-CD200R axis is involved in the regulation of antitumor response and in cancer evasion [5,28,29,30]. In AML an aberrant expression of CD200 has been associated with poor survival in all cytogenetic risk groups [18,31]. Coles et al. observed a reduction of activated natural killer cells, defective NK cytolytic activity, reduced CD4 Th1 memory and memory cytotoxic response, and high Treg frequency in cases of AML with CD200 expression, thus explaining the increased relapse risk and the worse survival among these patients [32,33,34]. We have observed an increase of myeloid precursors with suppressive activity, suggesting that the binding of CD200R on myeloid cells could play a role in the development of a leukemia-permissive micro-environment and in the reduction of the anti-infective immune response (personal data, unpublished).

In the present work we associate, for the first time, BCL2 and CD200 concomitant expression with a lower survival probability when compared to cases with isolated BCL2 or CD200 expression or DN. The negative impact of BCL2/CD200 co-expression on OS was also maintained in multivariate analysis, along with known prognostic factors such as advanced age, high WBC count (a surrogate of leukemic burden), secondary AML, and CD34 positivity. The combination of these five factors in a risk score based on their presence or absence defined five subgroups with very different survival probabilities.

The mechanism by which BCL2 synergizes with CD200 in affecting prognosis is far from being clarified. The deregulation of BCL2 proteins has been mostly associated with a survival advantage of neoplastic cells, but the increasing knowledge of the structural and functional diversity of BCL2 family members and their different cellular localizations has highlighted their involvement in cell functions other than apoptotic control [35]. In cancer cell lines BCL2 overexpression seems able to promote cell migration, increasing metastatic potential [36,37], by regulating Ca^2+^ homeostasis and by indirectly inducing the production of MMP-9, able to detach leukemic cells from their extracellular matrix [38,39,40]. Thus, double-positive BCL2/CD200 leukemic cells could take advantage not only of a higher intrinsic survival capacity but also an enhanced dissemination ability in a CD200-induced permissive micro-environment.

## Conclusion

In the era of targeted therapies, these data suggest the intriguing possibility of killing leukemic cells by normalizing the balance between anti- and pro-apoptotic activities of BCL2 family members, e.g., by using the BH3 mimetic venetoclax, and simultaneously restoring the antitumor immune activity by anti-CD200 antibodies blocking the CD200 pathway.

## Figures and Tables

**Table 1:**
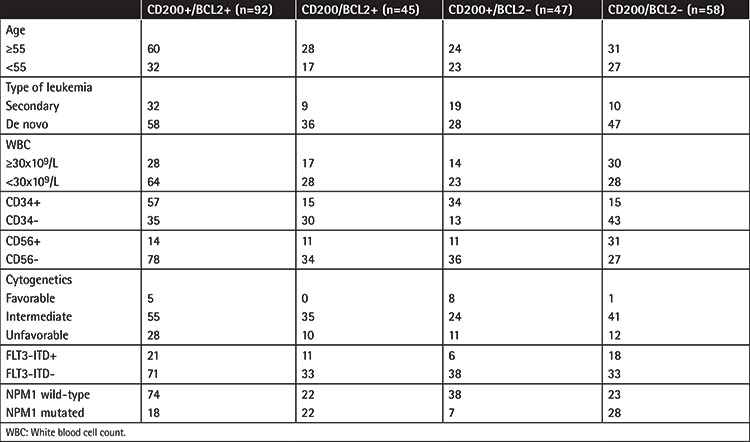
Patients’ characteristics according to CD200 positivity and BCL2 overexpression.

**Table 2:**
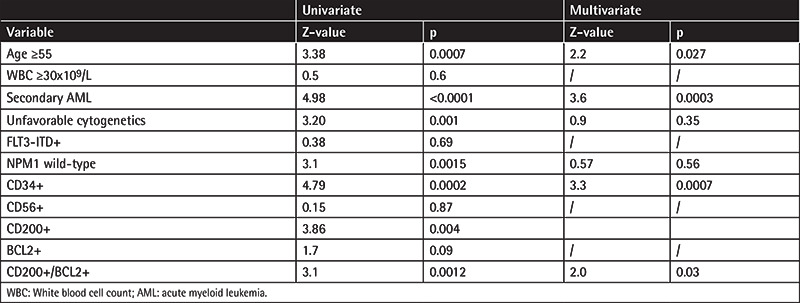
Factors associated with low remission rate in univariate and multivariate analysis. Univariate Multivariate

**Table 3:**
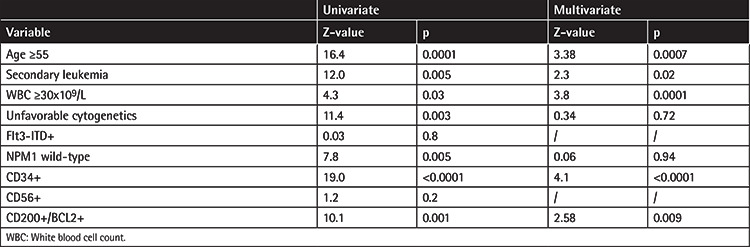
Univariate and multivariate analysis for factors affecting overall survival.
